# A preliminary study on the evaluation of left atrial function of rheumatoid arthritis by two dimensional speckle tracking imaging

**DOI:** 10.1038/s41598-021-00657-0

**Published:** 2021-11-02

**Authors:** Xiang Ji, Xia Zhang, Guojie Li

**Affiliations:** 1 Department of Ultrasound, Dafeng People’s Hospital Affiliated to Medical College of Yangzhou University, Yancheng, 224100 Jiangsu People’s Republic of China; 2 Department of Ultrasound, The First Affiliated Hospital of Wannan Medical College, 2 West Zheshan Road, Wuhu, 241001 Anhui People’s Republic of China

**Keywords:** Cardiology, Medical research, Rheumatology

## Abstract

To evaluate the changes of left atrial (LA) geometry and function in patients with rheumatoid arthritis (RA) by conventional echocardiography and two-dimensional speckle tracking imaging (2D-STI). We enrolled 46 RA patients with a duration of < 5 years as Group I, 40 RA patients with a duration of ≥ 5 years as Group II, and 40 normal subjects as the control group. Conventional echocardiography was conducted to measure traditional parameters. The LA strain during reservoir phase (LASr), LA strain during conduit phase (LAScd), LA strain during contraction phase (LASct), and LA global longitudinal strain (LAGLS) were obtained from 2D-STI. Related ultrasound results were compared. The LASct was significantly higher in Group I than in control group (P < 0.05). The LASr, LAScd, and LAGLS were significantly lower in Group I than in control group (all P < 0.05). The LASr, LAScd, LASct, and LAGLS were significantly lower in Group II than in control group and Group I (all P < 0.05). The function of LA impaired in RA patients, and the impairment aggravated with the clinical course of RA patients. 2D-STI technology can early and accurately evaluate the LA function of RA patients by evaluating LASr, LAScd, LASct, and LAGLS.

## Introduction

Rheumatoid arthritis (RA) is a chronic systemic inflammatory disease, which could damage joints, skin, eyes, lung, kidney, nervous system, gastrointestinal tract, heart, and blood vessels^1^. RA patients account for 0.5–1% of the world's population^2^. RA is an independent risk factor for cardiovascular disease^3^. The prevalence of cardiovascular disease in RA is very high, which is 2–5 times of normal subjects^4–6^. Even with no history of heart disease, RA patients are highly likely to have abnormal cardiac structure and function. The risk of cardiovascular death in RA patients is more than 50% higher than that in healthy persons^7,8^. Cardiac involvement is the main cause of death in RA patients^9^. Therefore, early detection of myocardial function in RA patients has important value for further clinical decision-making and reducing mortality because of the high incidence rate and the occult onset of cardiovascular complications in RA patients.

The structural and functional abnormalities of the left atrium occur early in cardiovascular diseases. Left atrial (LA) dysfunction is closely related to cardiovascular diseases such as atrial fibrillation^10,11^, stroke^12,13^, heart failure, and even death^14^. Hence, early detection of LA function in RA patients is of importance. However, there are few studies on the evaluation of LA function in RA patients till date.

Although angiography can accurately evaluate LA function, it is invasive. Real time three-dimensional echocardiography (RT-3DE) can noninvasively evaluate the structure and function of left atrium, however, the complete heart wall could not be observed for patients with significant heart enlargement because of the relatively low resolution and low image clarity of RT-3DE. Tissue Doppler imaging (TDI) can reflect the LA function by evaluating the LA strain, but it is angle-dependent^15^, and is easily affected by respiration and frame rate.

Two dimensional speckle tracking imaging (2D-STI) is a new technique developed in recent years. In this technique with angle-independence, by tracking the speckle patterns in the two-dimensional plane, the image-processing algorithm allows for better evaluation of global and local myocardial deformation^16,17^. 2D-STI has been used in the evaluation of many cardiovascular diseases, such as coronary heart disease and diabetes^18–21^. However, few studies have evaluated LA function by 2D-STI in RA patients till date.

In order to provide the basis for early intervention to prevent the occurrence of adverse cardiovascular events in RA patients, we evaluated the LA structure and function in RA patients by conventional echocardiography and 2D-STI.

## Methods

### Study population and control subjects

Eighty-six RA patients with normal left ventricular ejection fraction (LVEF) were selected in the first affiliated hospital of Wannan Medical College from September 2019 to September 2020. All patients were diagnosed by the same experienced rheumatologist and fulfilled the American College of Rheumatology/European League Against Rheumatism (ACR/EULAR) 2010 criteria^22^. RA patients were divided into two groups according to the clinical course of RA patients. Group I included 10 males and 36 females who had a mean duration of disease of < 5 years, and the patients in this group have a mean age of 44.25 years, with a standard deviation of 6.69 years, and their incident age ranged from 32 to 55 years. Group II included 11 males and 29 females who had a mean duration of disease of ≥ 5 years, and the patients in this group have a mean age of 44.61 years, with a standard deviation of 6.18 years, and their incident age ranged from 34 to 55 years. Forty healthy normal subjects matched in age, gender, heart rate (HR), and body surface area (BSA) were selected as control subjects. Control group included 10 males and 30 females, and the subjects in this group had a mean age of 43.04 years, with a standard deviation of 6.61 years, and their incident age ranged from 31 to 56 years. The exclusion criteria for this study were cardiac involvement, including valve diseases, congenital heart diseases, cardiomyopathy, coronary heart diseases, cardiac rhythm abnormalities, and pericardial effusion, etc.; extracardiac diseases that could lead to cardiac dysfunction, such as arterial hypertension, renal insufficiency, and diabetes, etc.; inability to follow commands during examinations; and technically poor acoustic window precluding satisfactory two dimensional echocardiographic imaging of left atrium. We excluded three patients with pericardial effusion, four patients with hypertension, three patients with valve diseases, and two patients with diabetes.

### Echocardiograms

Transthoracic two-dimensional echocardiography was performed in all participants by the utilize of Philips EPIQ 7C ultrasound system (Philips, Best, The Netherlands) equipped with a S5-1 probe with a frequency of 3.5–5 MHz. The left ventricular posterior wall thickness (LVPWT) at the end of diastole was obtained from the long axis view of left ventricle. LA minimum volume (LAV_min_), LA maximal volume (LAV_max_), and LA presystolic volume (LAV_prep_) were estimated using Simpson’s biplane method of disks in the four-and two-chamber views. The LVEF and left ventricular fractional shortening (LVFS) were also calculated by the biplane method of disks. The LA ejection fraction (LAEF) was calculated using the formula: (LAV_max _− LAV_min_)/LAV_max_. The LA active ejection fraction (LAAEF) was calculated using the formula: (LAV_pre_ − LAV_min_)/LA_pre_ × 100%. The LA passive ejection fraction (LAPEF) was calculated using the formula: (LAV_max_ − LAV_pre_)/LA_max_ × 100%. The LV mass was calculated using the Devereux formula^23^. The sex, age, height, weight, blood pressure, and heart rate of all the subjects were recorded. Body surface area (BSA) was calculated according to height and weight as follows: BSA (m^2^) = 0.0061 × height (cm) + 0.0128 × weight (kg)  − 0.1529. The routine LA parameters were standardized through BSA, and the standardized parameters of the LA maximal volume index (LAVI_max_), LA minimum volume index (LAVI_min_), LA presystolic volume index (LAVI_prep_), and left ventricular mass index (LVMI) were then obtained.

**Figure 1:**
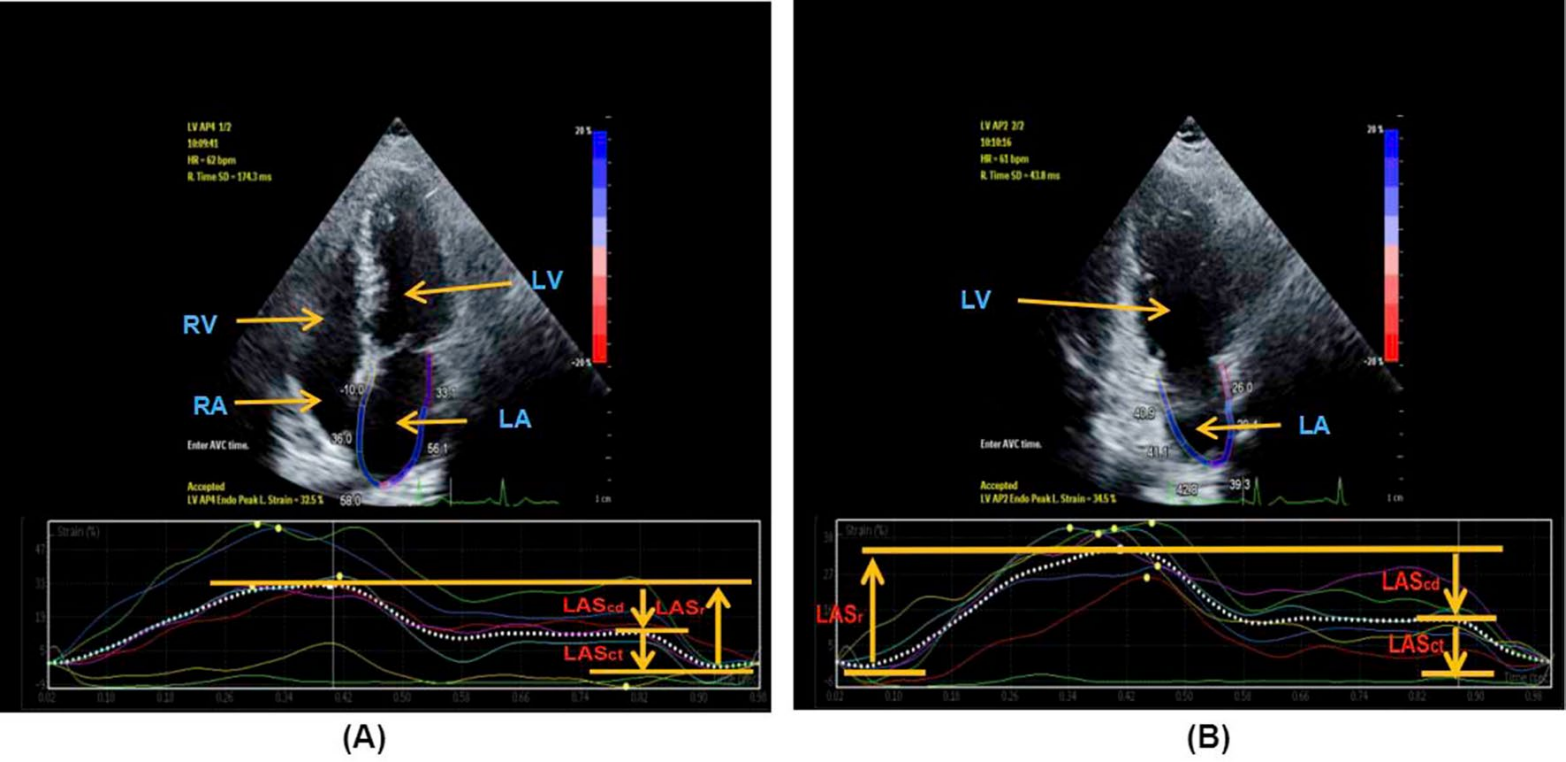
2D-STI parameters of left atrium in control group. 2D-STI parameters of left atrium obtained from apical four-chamber view in control group (**A**). 2D-STI parameters of left atrium obtained from apical two-chamber view in control group (**B**). *LAGLS* left atrial global longitudinal strain, *LASr* left atrial strain during reservoir phase, *LAScd* left atrial strain during conduit phase, *LASct* left atrial strain during contraction phase, *LV* left ventricle, *LA* left atrium, *RV* right ventricle, *RA* right atrium.

**Figure 2:**
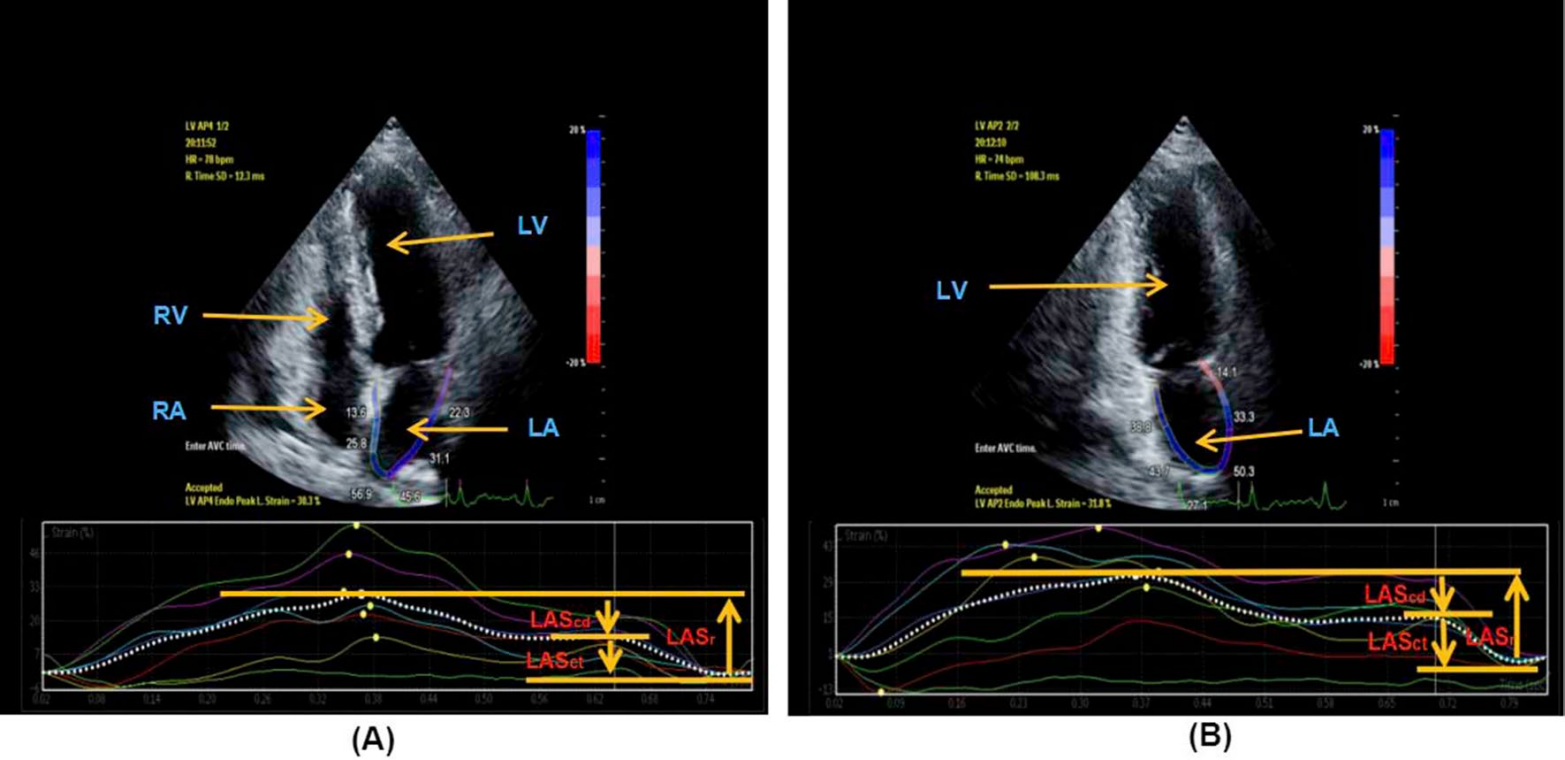
2D-STI parameters of left atrium in Group I. 2D-STI parameters of left atrium obtained from apical four-chamber view in Group I (**A**). 2D-STI parameters of left atrium obtained from apical two-chamber view in Group I (**B**). *LAGLS* left atrial global longitudinal strain, *LASr* left atrial strain during reservoir phase, *LAScd* left atrial strain during conduit phase, *LASct* left atrial strain during contraction phase, *LV* left ventricle, *LA* left atrium, *RV* right ventricle, *RA* right atrium.

**Figure 3:**
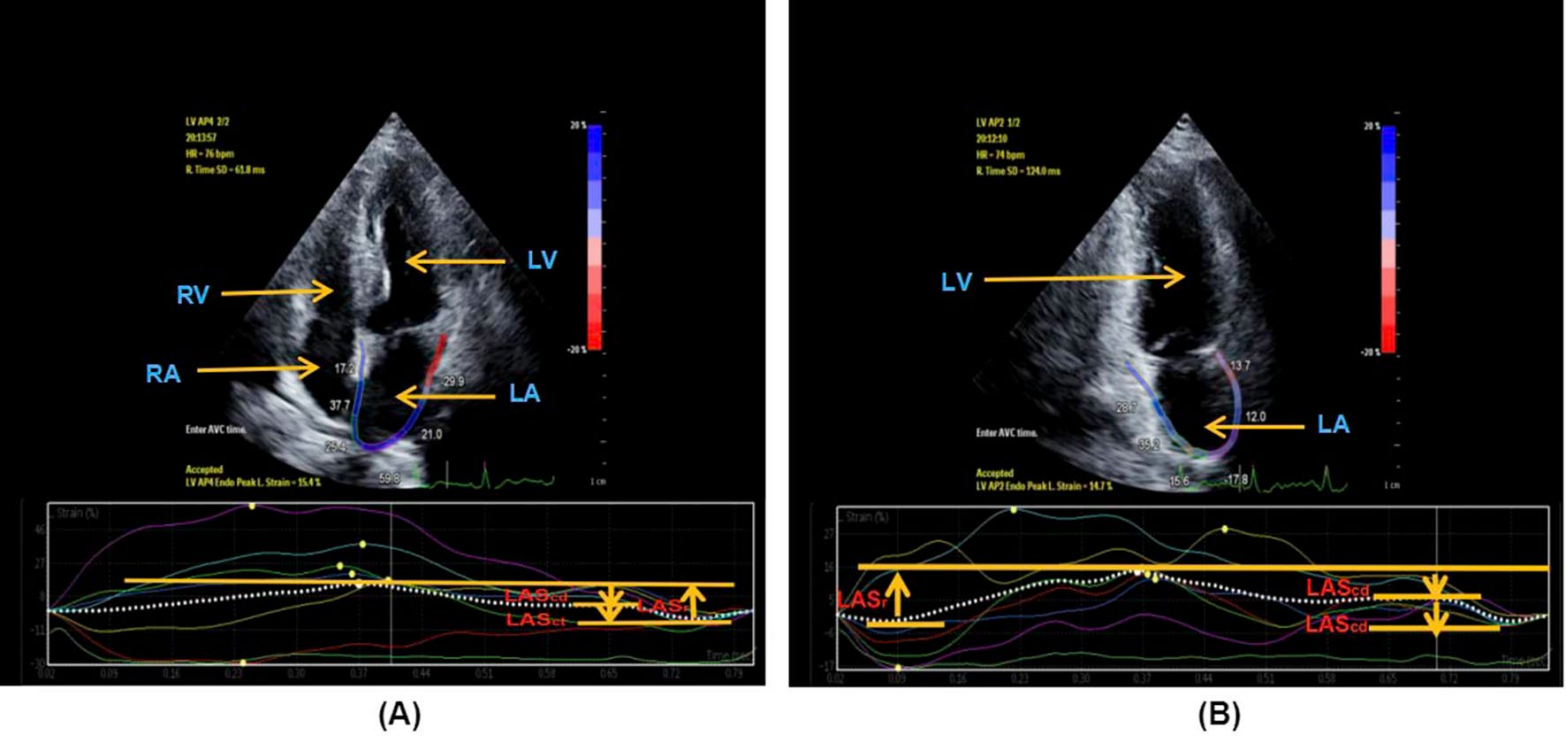
2D-STI parameters of left atrium in Group II. 2D-STI parameters of left atrium obtained from apical four-chamber view in Group II (**A**). 2D-STI parameters of left atrium obtained from apical two-chamber view in Group II (**B**). *LAGLS* left atrial global longitudinal strain, *LASr* left atrial strain during reservoir phase, *LAScd* left atrial strain during conduit phase, *LASct* left atrial strain during contraction phase, *LV* left ventricle, *LA* left atrium, *RV* right ventricle, *RA* right atrium.

The collected two-dimensional dynamic images were transferred to QLAB (version 10.5, Philips Medical System) workstation, and myocardial strain was analyzed in the interface of aCMQ program. According to the American Society of Echocardiography (ASE) and European Association of cardiovascular imaging (EACVI) recommended guidelines in 2018, an adjustable region of interest with a default width of 3 mm is set^24^. Two satisfactory plane views were selected, including apical two-and four-chamber views. The software automatically tracked the myocardial movement and outlined the endocardium. The unsatisfactory endocardium outlined by the software was then manually adjusted to wrap the region of interest around the whole myocardial layer. The strain value at mitral valve opening (corresponding to ECG U wave peak), the strain value at the onset of atrial contraction (corresponding to ECG P wave peak), the strain value ventricular end-diastole (corresponding to ECG S wave valley) in apical two- and four- chamber views, and the LA global strain (LAGLS) in each view were recorded. LASr = LA strain during reservoir phase, measured as difference of the strain value at mitral valve opening minus ventricular end-diastole (positive value). LAScd = LA strain during conduit phase, measured as difference of the strain value at the onset of atrial contraction minus mitral valve opening (negative value). LASct = LA strain during contraction phase, measured as difference of the strain value at ventricular end diastole minus onset of atrial contraction (negative value) (Figs. 1, 2, 3).

Thirty data sets were randomly selected to test the repeatability of LASr, LAScd, and LASct within and among observers. The intraobserver consistency test was analyzed by the same operator at least 1 week apart with 2D-STI software, and an another experienced operator was selected to analyze the data of 30 patients without knowing the results of the former. The two sets of results were then compared to test the consistency between the observers.

### Statistics

SPSS 21.0 software was used for statistical analysis. Continuous outcome variables were presented as mean ± standard deviation (SD), and non-normally distributed variables were reported as median and interquartile range (IQR). For the quantitative data with normal distribution and homogeneous variance, the independent samples t-test or one-way ANOVA was used for comparison between or among groups. Otherwise, Mann–Whitney U test or Kruskal–Wallis one-way analysis of variance was used for comparison between or among groups. The chi-square or Fisher’s exact test was used for the analysis of categorical data. Pearson^’^s correlation analysis was applied to show the correlation between two sets of data. Bland–Altman scatter plot was used to show the results of the repeatability tests of interobservers and intraobserver. A P value of < 0.05 was considered significant.

### Compliance with ethical standards

The study protocol was approved by the ethics committee of the First Affiliated Hospital of Wannan Medical College, and informed consent was obtained from each patient’s family.

### Ethical statement

All patients involved in this study gave their informed consent. Institutional review board approval of our hospital was obtained for this study. Informed consent was obtained from all participants included in the study and the photographs in which a patient or other person is identifiable have been published with written permission from that person. The persons are consenting to the publication of the photographs. The persons have no restrictions on the publication of photographs.

## Results

### Comparison of general data among groups

There were no significant differences in general data among the groups (all P > 0.05) (Table 1).

**Table 1:** Characteristics of all the subjects.

Parameter	Control	Group I	Group II	P-value
Age (years)	43.04 ± 6.61	44.25 ± 6.69	44.61 ± 6.18	0.53
BMI (kg/m^2^)	21.02 ± 1.96	20.78 ± 2.06	20.89 ± 1.78	0.84
Systolic pressure (mm/Hg)	115.32 ± 12.27	118.64 ± 13.30	118.72 ± 13.36	0.41
Diastolic pressure (mm/Hg)	74.35 ± 6.43	76.20 ± 6.54	77.43 ± 9.39	0.19
Female (%)	75.00	78.26	72.50	0.82
HR (bmp)	73.92 ± 5.10	74.30 ± 5.31	73.95 ± 5.31	0.93
Smoking (%)	5.0	6.5	7.5	0.90
Total cholesterol (mg/dL)	175.6 ± 18.1	172.4 ± 20.9	177.7 ± 19.8	0.46
HDL cholesterol (mg/dL)	52.0 ± 10.6	55.6 ± 11.2	55.2 ± 10.8	0.27
LDL cholesterol (mg/dL)	104.6 ± 10.9	103.9 ± 11.2	105.0 ± 11.7	0.89
**Medications**				
DMARDs (%)	–	89.1	95.0	0.32
NSAIDs (%)	–	37.0	50.0	0.22
Corticosteriod (%)	–	32.6	55.0^#^	0.04

Data are presented as mean ± standard deviation unless otherwise indicated.

*BMI* body mass index, *HR* heart rate, *HDL cholesterol* high density lipoprotein cholesterol; *LDL cholesterol* low density lipoprotein cholesterol, *DMARDs* disease modifying anti-rheumatic drugs, *NSAIDs* nonsteroidal anti-inflammatory drugs.

^#^Compared with group I, P < 0.05.

### Comparison of clinical and laboratory parameters of RA among groups

Among RA patients, there were no significant differences in rheumatoid factor (RF) and anti-cyclic peptide containing citrulline (Anti CCP) between the groups (both P > 0.05). The clinical course of RA patients was significantly longer in Group II than in Group I (P < 0.05). Among all the subjects, RF and Anti CCP were significantly higher in Group I and Group II than in control group (all P < 0.05) (Table 2).

**Table 2:** Clinical and laboratory parameters.

Parameter	Control	Group I	Group II	P-value
**Clinical**				
RA duration (years)	–	2.42 ± 1.13	7.21 ± 1.39^#^	< 0.001
**Laboratory**				
RF positive (%)	–	66	65	0.98
RF (IU/ml)	4.69 (2.87, 7.87)	138.34 (18.56, 178.61)*	131.28 (17.32, 183.81)*	< 0.001
Anti CCP positive (%)	–	62	60	0.62
Anti CCP (U/ml)	7.03 (4.70, 9.07)	180.45 (20.02, 300.31)*	186.33 (19.93, 296.90)*	< 0.001

Data are presented as mean ± standard deviation, median (interquartile range), or positive rate.

*RF* rheumatoid factor, *Anti CCP* anti-cyclic peptide containing citrulline.

*Compared with control group, P < 0.05.

^#^Compared with group I, P < 0.05.

### Comparison of conventional echocardiographic parameters among groups

Control group and Group I showed no significant differences in LVPWT, LVEF, and LVFS (all P > 0.05). Compared with the control group and Group I, there were no significant differences in LVEF and LVFS in Group II (all P > 0.05). The LVMI, E/e, LAVI_max_, LAVI_prep_, LAVI_min_, and LAAEF were significantly higher and the e, LAEF, and LAPEF were significantly lower in Group I than in control group (all P < 0.05). The LVMI, E/e, LAVI_max_, LAVI_prep_, and LAVI_min_ were significantly higher and the e, LAEF, LAPEF, and LAAEF were significantly lower in Group II than in control group and Group I (all P < 0.05) (Table 3).

**Table 3:** Conventional echocardiographic parameters.

Parameter	Control	Group I	Group II	P-value
LAVI_max_ (ml/m^2^)	24.11 ± 2.41	30.41 ± 2.82*	38.56 ± 2.70*^#^	< 0.001
LAVI_min_ (ml/m^2^)	10.13 ± 1.90	14.72 ± 1.79*	24.21 ± 2.99*^#^	< 0.001
LAVI_pre_ (ml/m^2^)	13.31 ± 2.11	20.82 ± 2.44*	29.80 ± 2.90*^#^	< 0.001
LAEF (%)	58.18 ± 5.45	51.72 ± 3.00*	37.23 ± 7.05*^#^	< 0.001
LAPEF (%)	45.00 ± 5.46	31.60 ± 3.96*	22.68 ± 5.66*^#^	< 0.001
LAAEF (%)	25.34 ± 5.93	29.46 ± 4.40*	18.83 ± 4.61*^#^	< 0.001
E/e	7.70 ± 1.32	12.26 ± 2.06*	15.56 ± 1.60*^#^	< 0.001
e	10.20 ± 1.79	7.04 ± 1.15*	4.82 ± 1.21*^#^	< 0.001
LVEF (%)	66.27 ± 2.44	65.93 ± 2.16	65.56 ± 2.08	0.36
LVFS (%)	36.96 ± 1.31	36.65 ± 1.17	36.45 ± 1.21	0.18
LVMI (g/m^2^)	78.68 ± 8.89	85.11 ± 13.76*	97.83 ± 19.03*^#^	< 0.001
LVPWT (mm)	8.76 ± 0.21	8.82 ± 0.23	12.52 ± 0.22*^#^	< 0.001

Data are presented as mean ± standard deviation.

*LAVI*_*max*_ left atrial maximal volume index, *LAVI*_*min*_ left atrial minimum volume index, *LAVI*_*pre*_ left atrial presystolic volume index, *LAEF* left atrial ejection fraction, *LAPEF* left atrial passive ejection fraction, *LAAEF* left atrial active ejection fraction, *LVEF* left ventricular ejection fraction, *LVFS* left ventricular fractional shortening, *LVMI* left ventricular mass index, *LVPWT* left ventricular posterior wall thickness.

*Compared with control group, P < 0.05.

^#^Compared with group I, P < 0.05.

### Comparison of strain values among groups

The LAsct was significantly higher and the LASr, LAScd, and LAGLS were significantly lower in Group I than in control group (all P < 0.05). The LASr, LAScd, LASct, and LAGLS were significantly lower in Group II than in control group and Group I (all P < 0.05) (Table 4).

**Table 4:** Strain analysis.

Parameter	Control	Group I	Group II	P-value
LASr (%)	38.52 ± 3.17	28.82 ± 2.17*	18.42 ± 1.76*^#^	< 0.001
LAScd (%)	− 22.58 ± 1.91	− 15.59 ± 1.62*	− 9.46 ± 2.60*^#^	< 0.001
LASct (%)	− 12.97 ± 1.53	-15.24 ± 4.70*	− 6.94 ± 1.49*^#^	< 0.001
LAGLS (%)	34.56 ± 3.61	28.06 ± 2.40*	17.38 ± 1.34*^#^	< 0.001

Data are presented as mean ± standard deviation.

*LASr* strain during reservoir phase, *LAScd* strain during conduit phase, *LASct* strain during contraction phase, *LAGLS* left atrial global longitudinal strain.

*Compared with control group, P < 0.05.

^#^Compared with group I, P < 0.05.

### Correlation analysis

Among RA patients, no significant correlation was found between the LAGLS and inflammatory markers, including the RF level and anti-CCP level (r = − 0.12, − 0.10, respectively, both P > 0.05) (Table 5). A significant correlation was found between the clinical course of RA patients and the parameters of LA function, including LAGLS, LASr, absolute value of LAScd, absolute value of LASct, LAEF, LAPEF, and LAAEF (r = − 0.93, − 0.94, − 0.90, − 0.73, − 0.93, − 0.81, and − 0.81, respectively; all P < 0.05) (Table 6).

**Table 5:** Association between LAGLS and inflammation related indexes in RA patients.

Variable	Correlation coefficient	P-value
RF (IU/ml)	− 0.12	0.28
Anti CCP (U/ml)	− 0.10	0.37

*RF* rheumatoid factor, *Anti CCP* anti-cyclic peptide containing citrulline.

**Table 6:** Association between the clinical course and LA function parameters in RA patients.

Variable	Correlation coefficient	P-value
LAGLS (%)	− 0.93	< 0.001
LASr (%)	− 0.94	< 0.001
Absolute value of LAScd (%)	− 0.90	< 0.001
Absolute value of LASct (%)	− 0.73	< 0.001
LAEF (%)	− 0.93	< 0.001
LAPEF (%)	− 0.81	< 0.001
LAAEF (%)	− 0.81	< 0.001

*LAGLS* left atrial global longitudinal strain, *LASr* left atrial strain during reservoir phase, *LAScd* left atrial strain during conduit phase, *LASct* left atrial strain during contraction phase, *LAEF* left atrial ejection fraction, *LAPEF* left atrial passive ejection fraction, *LAAEF* left atrial active ejection fraction.

**Figure 4:**
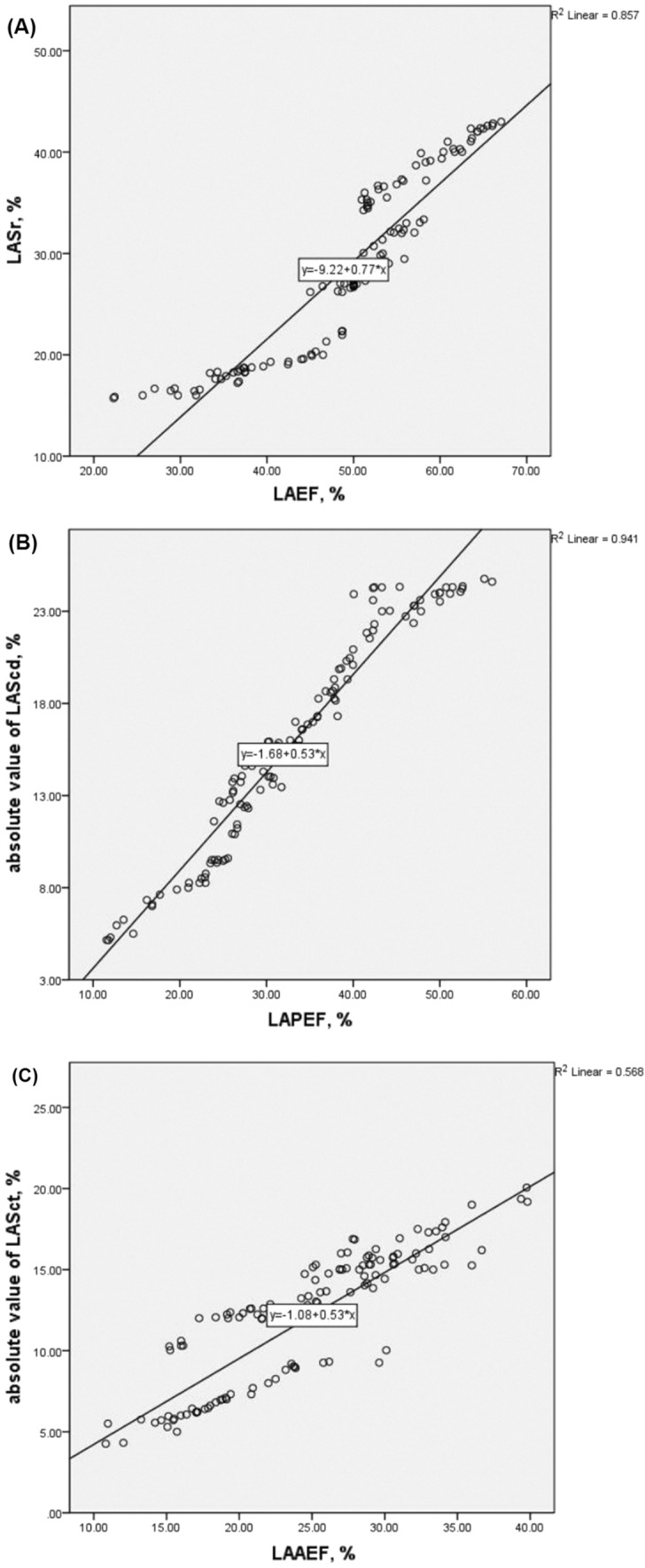
Linear correlations between LA strain parameters and the parameters of traditional LA function. Linear correlation between LASr and LAEF (**A**). Linear correlation between absolute value of LAScd and LAPEF (**B**). Linear correlation between absolute value of LASct and LAAEF (**C**).

In all the subjects, LAEF was positively correlated with LASr (r = 0.93, P < 0.05), LAPEF was positively correlated with the absolute value of LAScd (r = − 0.97, P < 0.05), and LAAEF was positively correlated with the absolute value of LASct (r = − 0.75, P < 0.05) (Fig. 4).

### Repeatability analysis

**Figure 5:**
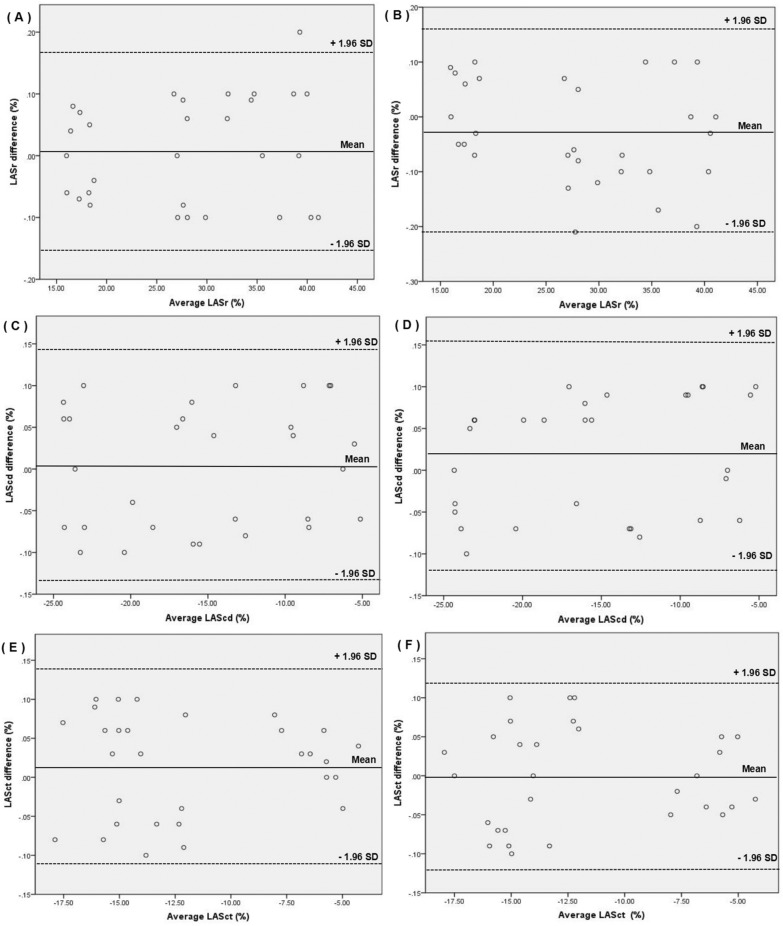
Bland–Altman analyses. Bland–Altman analysis of LASr obtained by 2D-STI showed that there was a high consistency in interobservers (**A**). Bland–Altman analysis of LASr obtained by 2D-STI showed that there was a high consistency in intraobserver (**B**). Bland–Altman analysis of LAScd obtained by 2D-STI showed that there was a high consistency in interobservers (**C**). Bland–Altman analysis of LAScd obtained by 2D-STI showed that there was a high consistency in intraobserver (**D**). Bland–Altman analysis of LASct obtained by 2D-STI showed that there was a high consistency in interobservers (**E**). Bland–Altman analysis of LASct obtained by 2D-STI showed that there was a high consistency in intraobserver (**F**).

Bland–Altman analyses of the LA strain parameters obtained by 2D-STI showed high interobserver and intraobserver consistency (Fig. 5).

## Discussion

Left atrium is a structure connecting pulmonary vein and left ventricle, which plays an important role in maintaining normal cardiovascular function. LA function may be helpful for the evaluation of left ventricular diastolic function^25^. The size of left atrium is a predictor of stroke and atrial fibrillation^26^. In different phases of the cardiac cycle including systole, early diastole, and late diastole. LA function serves as reservoir function, conduit function, and contractile function respectively. LA relaxation and compliance could be reflected by LA reservoir function. LV diastolic function is closely related to LA conduit function. Intrinsic LA contractility and LV end-diastolic compliance and pressure could be reflected by LA contractile function^27^. Some studies have shown the LA enlargement in RA patients. However, the evaluation of LA strain function, the correlations between biochemical parameters of RA patients and LA strain parameters, and the correlations between the parameters of LA function and the clinical course of RA patients were rarely mentioned in literature to date.

Conventional echocardiography has been widely used in the evaluation of LA function and structure, but it has some limitations. LA enlargement is an independent predictor of cardiovascular risk events, but it is affected by gender, age, height, weight, and other factors. Mitral flow spectrum can help to diagnose atrial fibrillation, but it is affected by heart rate, LA load, and the position of sampling line. TDI can also be used to evaluate the LA strain function, but it is angle-independent. RT-3DE can reflect the structure and function of left atrium more objectively than conventional echocardiography, but low resolution and image clarity could reduce the accuracy of RT-3DE measurement.

2D-STI is a practical technique with high accuracy, sensitivity, and specificity. Strain echocardiography has played a key role in the assessment of cardiac diseases^19^. The image-processing algorithm could give a better evaluation of global and local myocardial deformation by tracking the speckle patterns in 2-D plane in 2D-STI^28,29^. 2D-STI is superior to strain imaging by TDI because of the independence of the Doppler angle of incidence^30^. Several studies have well evaluated the left ventricular deformation in RA patients by 2D-STI^31–34^. Although 2D-STI was initially used to evaluated left ventricular function, it can also be used in the assessment of atrial function. Global longitudinal deformation parameters of left atrium were recommended since the LA myocardium is thin and the sufficient detail could usually not be resolved by the echocardiographic images for reliable local tracking. The LA global deformation could be accurately assessed by the utilize of 2D-STI. Some studies have confirmed the usefulness of 2D-STI in evaluating LA dysfunction^35–37^. We assessed the LASr, LAScd, LASct, and LAGLS by 2D-STI in our study and found remarkable differences between healthy persons and RA patients.

In this study, the Group II was significantly treated by corticosteroids. Our results revealed that the LV geometry may be changed in RA patients and aggravated with the prolongation of the RA disease duration, which is in accordance with previous study^38^. Chronic inflammation and long term use of immunosuppressive hormone drugs may contribute to the development of LV remodeling and LA enlargement. According to Frank Starling mechanism, the contractility of LA myocardium increases with the expansion of left atrium so as to maintain sufficient filling pressure. The long-term increase of LA pressure will lead to the further LA enlargement.

Our results indicated that the left ventricular diastolic function impaired in RA patients, which is in accordance with previous study^39,40^. Inflammatory factors, metabolic disorders, and drug effects in RA patients could bring increased left ventricular pressure and LA afterload, contributing to the LA enlargement. The less physical activity and the persistent inflammation in RA patients may lead to diastolic dysfunction. With the prolongation of RA duration, the left atrium further expanded and the left ventricular remodeling aggravated, resulting in the progression of diastolic dysfunction.

To the best of our knowledge, this is the first time to evaluate LASr, LAScd, and LASct in RA patients. These parameters could more specifically reflect the LA function of RA patients. Our results were in line with a study in patients with RA by Çetin et al.^41^ who demonstrated a reduction of LAGLS in RA patients. LAEF, LAPEF, and LAAEF reflect the LA reserve function, conduit function, and booster pump function respectively. Our findings showed that the LA strain parameters were positively correlated with the traditional LA function parameters. Additionally, the results of this study also showed that impaired LA function was associated with the clinical course of RA patients. Left ventricular hypertrophy and diastolic dysfunction may be caused by the less physical activity and the persistent inflammation in RA patients, which can lead to the impairment of LAEF, LAPEF, LAGLS, LASr, and LAScd. In order to maintain the stroke volume, the contractility of LA myocardium increases with the expansion of left atrium, which leads to an increase in LASct and LAAEF in Group I. With the prolongation of RA duration, long-term less physical activity, persistent inflammation, and hormone therapies, which have immunosuppressive effects, may lead to more advanced coronary artery disease and further LA enlargement and thus lead to the further decrease of LAEF, LAPEF, LAGLS, LASr, and LAScd. The overstretch of LA myocardium may cause the impairment of LAAEF and LASct. Moreover, the phenomenon that the inflammatory damage of RA is accumulated over the duration of inflammation and the inflammatory factors reflect the current level of disease activity and inflammation may result in no correlation between LAGLS and inflammatory markers. This study also indicated the good reproducibility of 2D-STI in the measurement of LASr, LAScd, LASct, and LAGLS.

Major limitation of the presented study is a single centre experience with small number of patients. Further multicenter studies of larger samples of RA patients are needed to confirm our findings. In addition, the findings of the study cannot be applied on patients with histories of cardiac involvements who were excluded from our study. Finally, we had not used cardiac magnetic resonance (CMR) as this modality had not yet been developed in our hospital. In future study, we will apply this technique to detect inflammation in the heart.

## Conclusions

In RA patients with no clinical cardiovascular disease, the LA function is associated with the RA duration. 2D-STI is a convenient, accuracy, reproducible, and valuable method for assessing LA function in RA patients, which may be an important part of clinical judgment.

## References

[1] Smolen JS, Aletaha D, Mclinnes IB (2016). Rheumatoid arthritis. Lancet.

[2] Scott DL, Steer S (2007). The course of established rheumatoid arthritis. Best Pract. Res. Clin. Rheumatol..

[3] Corrales A (2015). Carotid artery plaque in women with rheumatoid arthritis and low estimated cardiovascular disease risk: A cross-sectional study. Arthritis Res. Ther..

[4] Atzeni F, Sarzi-Puttini P (2007). Early rheumatoid arthritis. Reumatismo.

[5] Cioffi G (2016). Combined circumferential andlongitudinal left ventricular systolic dysfunction in patients with rheumatoid arthritis without overt cardiac disease. J. Am. Soc. Echocardiogr..

[6] Nicola PJ (2006). Contribution of congestive heart failure and ischemic heart disease to excess mortality in rheumatoid arthritis. Arthritis Rheum..

[7] Curtis JR (2018). Is rheumatoid arthritis a cardiovascular risk-equivalent to diabetes mellitus?. Arthritis Care Res. (Hoboken).

[8] Aviña-Zubieta JA (2008). Risk of cardiovascular mortality in patients with rheumatoid arthritis: A meta-analysis of observational studies. Arthritis Rheum..

[9] Buleu F, Sirbu E, Caraba A, Dragan S (2019). Heart involvement in inflammatory rheumatic diseases: A systematic literature review. Medicina (Kaunas).

[10] Psaty BM (1997). Incidence of and risk factors for atrial fibrillation in older adults. Circulation.

[11] Leibson CL (2001). Left atrial volume: Important risk marker of incident atrial fibrillation in 1655 older men and women. Mayo Clin. Proc..

[12] Benjamin EJ, D'Agostino RB, Belanger AJ, Wolf PA, Levy D (1995). Left atrial size and the risk of stroke and death. The Framingham Heart Study. Circulation.

[13] Barnes ME (2004). Left atrial volume in the prediction of first ischemic stroke in an elderly cohort without atrial fibrillation. Mayo Clin. Proc..

[14] Essayagh B (2021). Left atrial dysfunction as marker of poor outcome in patients with hypertrophic cardiomyopathy. Arch Cardiovasc. Dis..

[15] Hunziker PR (1999). Regional wall motion assessment in stress echocardiography by tissue Doppler bull's-eyes. J. Am. Soc. Echocardiogr..

[16] Mcleod G (2018). Echocardiography in congenital heart disease. Prog. Cardiovasc. Dis..

[17] Guerra F (2016). Speckle-tracking global longitudinal strain as an early predictor of cardiotoxicity in breast carcinoma. Support Care Cancer..

[18] Yan P (2012). Left atrial and right atrial deformation in patients with coronary artery disease: A velocity vector imaging-based study. PLoS ONE.

[19] Liu YY (2011). Evaluation of left atrial function in patients with coronary artery disease by two-dimensional strain and strain rate imaging. Echocardiography.

[20] Nagueh SF (2016). Recommendations for the evaluation of left ventricular diastolic function by echocardiography: An update from the American Society of Echocardiography and the European Association of Cardiovascular Imaging. J. Am. Soc. Echocardiogr..

[21] Mokotedi L (2020). Associations of inflammatory markers with impaired left ventricular diastolic and systolic function in collagen-induced arthritis. PLoS ONE.

[22] Aletaha D (2010). 2010 rheumatoid arthritis classification criteria: An American College of Rheumatology/European League Against Rheumatism Collaborative Initiative. Ann. Rheum. Dis..

[23] Devereux RB (1986). Echocardiographic assessment of leftventricular hypertrophy: Comparison to necropsy findings. Am. J. Cardiol..

[24] Badano LP (2018). Standardization of left atrial, right ventricular, and right atrial deformation imaging using two-dimensional speckle tracking echocardiography: A consensus document of the EACVI/ASE/Industry Task Force to standardize deformation imaging. Eur. Heart J. Cardiovasc. Imaging.

[25] Thomas L, Marwick TH, Popescu BA, Donal E, Badano LP (2019). Left atrial structure and function, and left ventricular diastolic dysfunction: JACC state-of-the-art review. J. Am. Coll. Cardiol..

[26] Ay B (2015). Chronic inhibition of tumor necrosis factor-α with infliximab improves myocardial deformation in parallel with aortic elasticity in rheumatoid arthritis. Turk. Kardiyol. Dern. Ars..

[27] Tadic M, Cuspidi C (2021). Left atrial function in diabetes: Does it help?. Acta Diabetol..

[28] Muranaka A (2009). Quantitative assessment of left ventricular and left atrial functions by strain rate imaging in diabetic patients with and without hypertension. Echocardiography.

[29] Mondillo S (2011). Early detection of left atrial strain abnormalities by speckle tracking in hypertensive anddiabetic patients with normal left atrial size. J. Am. Soc. Echocardiogr..

[30] Sade LE, Gorcsan J, Severyn DA, Edelman K, Katz WE (2005). Usefulness of angle corrected tissue Doppler to assess segmental left ventricular function during dobutamine stress echocardiography in patients with and without coronary artery disease. Am. J. Cardiol..

[31] Løgstrup BB (2017). Left ventricular function at two-year followup in treatment-naive rheumatoid arthritis patients is associated with anti-cyclic citrullinated peptide antibody status: a cohort study. Scand. J. Rheumatol..

[32] Benacka O (2017). Speckle tracking can detect subclinical myocardial dysfunction in rheumatoid arthritis patients. Bratisl. Lek. Listy..

[33] Hanvivadhanakul P, Buakhamsri A (2019). Disease activity is associated with LV dysfunction in rheumatoid arthritis patients without clinical cardiovascular disease. Adv. Rheumatol..

[34] Atzeni F, Gianturco L, Boccassini L, Sarzi-Puttini P, Bonitta G, Turiel M (2019). Noninvasive imaging methods for evaluating cardiovascular involvement in patients with rheumatoid arthritis before and after anti-TNF drug treatment. Future Sci OA..

[35] Mohseni-Badalabadi R, Mehrabi-Pari S, Hosseinsabet A (2020). Evaluation of the left atrial function by two-dimensional speckle-tracking echocardiography in diabetic patients with obesity. Int. J. Cardiovasc. Imaging.

[36] Lenart-Migdalska A (2019). Assessment of left atrial function in patients with paroxysmal, persistent, and permanent atrial fibrillation using two-dimensional strain. J. Atr. Fibrillation.

[37] Cao S, Zhou Q, Chen JL, Hu B, Guo RQ (2016). The differences in left atrial function between ischemic and idiopathic dilated cardiomyopathy patients: A two-dimensional speckle tracking imaging study. J. Clin. Ultrasound.

[38] Oyama JI, Node K (2019). Rheumatoid arthritis and vascular failure—Rheumatoid arthritis is a risk factor for cardiovascular disease. Intern Med..

[39] Ayyildiz YO (2015). Effect of long-term TNF-α inhibition with infliximab on left ventricular torsion in patients with rheumatoid arthritis. Hellenic J. Cardiol..

[40] Fatma E (2015). Epicardial fat thickness in patients with rheumatoid arthritis. Afr. Health Sci..

[41] Çetin S (2014). Infliximab, an anti-TNF-alpha agent, improves left atrial abnormalities in patients with rheumatoid arthritis: Preliminary results. Cardiovasc. J. Afr..

